# Receptor hyper-flexibility of human bitter receptor TAS2R4 revealed by cryo-EM structures in apo and tripeptide-bound states

**DOI:** 10.1038/s41421-026-00895-4

**Published:** 2026-05-13

**Authors:** Yuxia Qian, Ming Tan, Zishao Ouyang, Sheng Ye, Jian Wu, Anna Qiao

**Affiliations:** 1 Tianjin Key Laboratory of Function and Application of Biological Macromolecular Structures, Faculty of Medicine, School of Life Sciences, Tianjin University, Tianjin, China; 2 Shanghai Institute of Precision Medicine, Ninth People’s Hospital, Shanghai Jiao Tong University School of Medicine, Shanghai, China; 3 State Key Laboratory of Synthetic Biology, Tianjin University, Tianjin, China; 4 Frontiers Science Center for Synthetic Biology (Ministry of Education), Tianjin University, Tianjin, China; 5 Life Sciences Institute, Zhejiang University, Hangzhou, Zhejiang China

**Keywords:** Cryoelectron microscopy, Molecular biology

Dear Editor,

Human bitter taste receptors (TAS2Rs) represent a unique class of G protein-coupled receptors (GPCRs) responsible for detecting a vast array of chemically diverse bitter compounds^1,2^. Unlike the more specialized sweet or umami receptors, ~26 members of the human TAS2R (hTAS2R) family must balance broad-spectrum recognition with fine-tuned signal transduction^3^. Among these, TAS2R4 stands out as an intermediately tuned receptor capable of responding to structurally distinct agonists, including various short-chain bitter peptides found in fermented foods (Supplementary Fig. S1)^4,5^. Despite the recent publications of TAS2R structural data^6–8^, the dynamic transition from an apo receptor to an active signaling complex remains largely elusive. Here, we present the cryo-electron microscopy (cryo-EM) structures of TAS2R4 in complex with mini-G protein gustducin (miniG_gust_) in both apo and tripeptide ^1^WWW^3^ (3W)-bound states (Fig. 1a–d). Our findings reveal a remarkable degree of receptor hyper-flexibility and a distinct activation mechanism that challenges current paradigms of class T GPCR signaling.

**Fig. 1:**
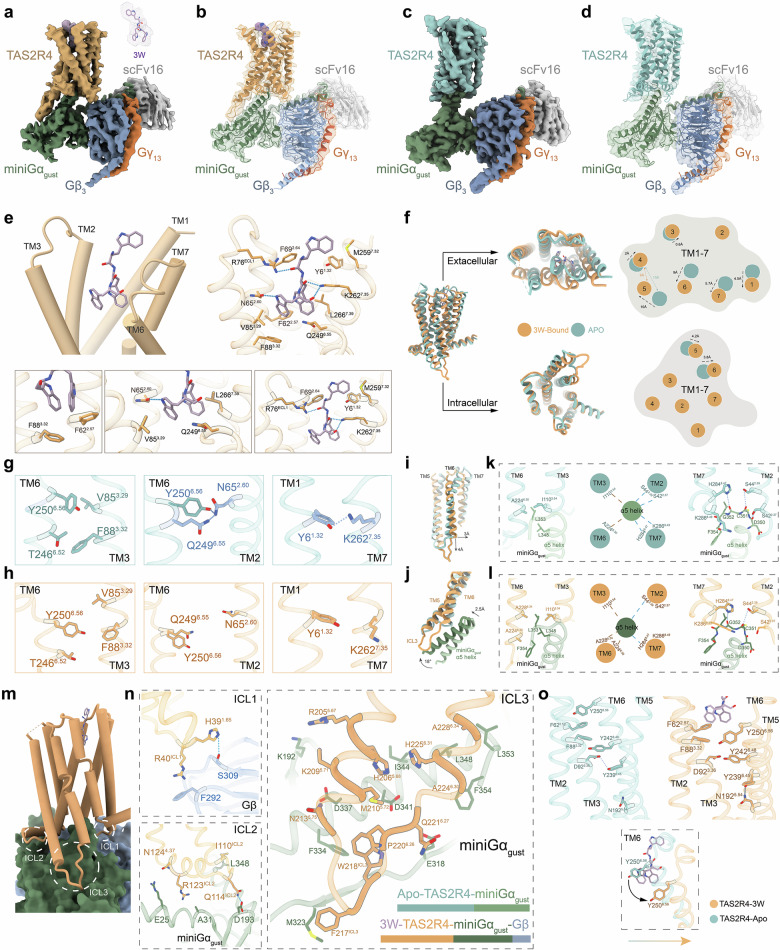
Cryo-EM structures of human bitter receptor TAS2R4 in apo and tripeptide-bound states. **a** Cryo-EM density map of 3W–TAS2R4–miniGα_gust_ complex. The density for 3W is clearly resolved in the ligand-binding pocket. **b** Cartoon representation of TAS2R4–miniGα_gust_ complex in the 3W-bound state, showing the transmembrane domain of TAS2R4 (orange), miniGα_gust_ (green), Gβ_3_ (blue), Gγ_13_ (brown), and scFv16 (gray), with 3W represented in purple. **c** Cryo-EM density map of apo–TAS2R4–miniGα_gust_ complex. **d** Cartoon representation of apo–TAS2R4–miniGα_gust_ complex, showing the transmembrane domain of TAS2R4 (cyan), miniGα_gust_ (green), Gβ_3_ (blue), Gγ_13_ (brown), and scFv16 (gray). **e** The overall illustrations of the interaction of 3W with TAS2R4, highlighting the key TMs (TM1, TM2, TM3, TM6, and TM7) involved in forming the orthosteric site (top left), with the key residues shown as sticks (top right); and close-up illustrations of key interactions in the binding pocket, highlighting the bottom (botton left), wall (botton middle), and extracellular edge (botton right) of the orthosteric site. **f** Structural comparison of TAS2R4 in the apo (cyan) and 3W-bound (orange) states. Left: side view of the superimposed structures. Right: views from the extracellular and intracellular perspectives, illustrating the movements of TMs (TM1–TM7) between the apo and 3W-bound states. **g** Key stabilizing interactions in the apo state. Residues involved in hydrogen bonding are shown with their side chains in blue. **h** Structural rearrangement of the residues shown in **g** after 3W binding. **i** Movement of TM6 in the 3W-bound state relative to the apo state. **j** Superimposed structures of apo and 3W-bound TAS2R4–miniGα_gust_ complexes emphasize the lateral (2.5 Å) and rotational (18°) movements of the α5 helix of miniGα_gust_ upon ligand binding. **k**, **l** Interaction diagrams between the α5 helix of miniGα_gust_ and TAS2R4 in the apo (**k**) and 3W-bound (**l**) states. Left: interactions with TM3 and TM6. Middle: simplified intracellular view illustrating the rearrangement of key interactions, where blue dashed lines represent polar interactions and brown dashed lines represent hydrophobic interactions. Right: interactions with TM2 and TM7. **m** Overall view of the interactions between TAS2R4 ICLs (ICL1, ICL2, ICL3) and G protein subunits (miniGα_gust_ and Gβ) in the 3W-bound state. **n** Close-up views of specific interactions between TAS2R4 ICLs (ICL1, ICL2, ICL3) and G protein subunits (miniGα_gust_ and Gβ) in the 3W-bound state. **o** Conformations of key residues involved in TAS2R4 activation in the apo and 3W-bound states. Upon 3W entering the binding pocket, Y250^6.56^ undergoes a significant rotation, swinging outward to accommodate the ligand. Conformational changes of the same residues upon 3W binding are shown.

The tripeptide 3 W is one of the most potent known agonists for TAS2R4 (Supplementary Fig. S3a). We first confirmed its functional activity using an intracellular calcium mobilization assay, yielding an EC_50_ value of 7.5 ± 0.1 μM (Supplementary Fig. S3b). To capture the structural snapshots of activation, we co-expressed TAS2R4 with a heterotrimeric G protein complex (miniGα_gust_ and Gβ_3_γ_13_) stabilized by the single-chain variable fragment scFv16. The resulting structures of the apo and 3W-bound TAS2R4–G protein complexes were determined at resolutions of 3.2 Å and 3.0 Å, respectively (Supplementary Fig. S2 and Table S1).

A striking feature of the TAS2R4 architecture is the high flexibility of extracellular loop 2 (ECL2) in both the apo and active states (Supplementary Fig. S4a–c, f). In canonical class A GPCRs, such as β2-adrenoceptor, ECL2 typically forms a well-defined “lid” or structured element that facilitates ligand binding and protects the orthosteric pocket (Supplementary Fig. S4d)^9^. The flexibility of ECL2 in TAS2R4 is consistent with two specific sequence motifs of TAS2Rs. First, the highly conserved disulfide bond observed in class A family GPCR structures between C^3.25^ (Ballesteros–Weinstein numbering used in superscript) and a cysteine residue in ECL2, which effectively ties ECL2 to the transmembrane core, is not present in TAS2Rs (Supplementary Fig. S5). Second, all hTAS2Rs contain a conserved consensus sequence for Asn-linked glycosylation (Asn–Xaa–Ser/Thr) within ECL2 (Supplementary Fig. S5)^10^. The bulky glycan chains may introduce steric hindrance near the orthosteric pocket, preventing ECL2 from adopting a fixed conformation while favoring an open, accessible state with multiple conformational microstates. Together, these two features afford TAS2R4 a uniquely “wide-open” orthosteric vestibule. This architectural flexibility may be essential for accommodating bulky, extended agonists like the 3W tripeptide. Interestingly, while TAS2R46 was recently shown to possess an ordered ECL2 in its apo state that partially occupies the pocket (Supplementary Fig. S4e)^8^, our TAS2R4 structure demonstrates that bitter receptors can exist in a flexible state even in the absence of a ligand.

The 3W-bound structure reveals that the tripeptide adopts an extended conformation within the orthosteric pocket, interacting with residues across transmembrane helix 1 (TM1), TM2, TM3, TM6, and TM7 (Fig. 1e). Two phenylalanine residues, F62^2.57^ and F88^3.32^, form the bottom, while N65^2.60^, V85^3.29^, Q249^6.58^, and L266^7.39^, form the wall of the orthosteric pocket, together holding two tryptophan side chains of 3W (Fig. 1e). The N-terminal tryptophan of 3W extends to the extracellular side, forming interactions with Y6^1.32^, F69^2.64^, R76^ECL1^, M259^7.32^, and K262^7.35^ (Fig. 1e).

Our comprehensive alanine-scanning mutagenesis in combination with an intracellular calcium mobilization assay highlights two key points. First, residues that interact with the hydrophobic side chain of 3W are crucial for binding, as alanine mutations in these hydrophobic residues, including F62^2.57^A, F88^3.32^A, V85^3.29^A, Y6^1.32^A, F69^2.64^A, either abolished or significantly reduced 3W activity on TAS2R4 (Supplementary Fig. S6a, b and Table S2). Second, four hydrogen bonds between R76^ECL1^ and the carbonyl oxygen of W^1^, K262^7.35^ and the carbonyl oxygens of W^2^ and W^3^, and N65^2.60^ and the side-chain amide of W^3^, are essential for ligand binding, as mutants N65^2.60^A and R76^ECL1^A abolished, and K262^7.35^A significantly reduced 3W activity on TAS2R4 (Supplementary Fig. S6a, b and Table S2). Notably, K262^7.35^ of TAS2R4 was previously identified to play important roles in interacting with agonists or antagonists^10^. And W88^3.32^ of TAS2R46, the corresponding residue of F88^3.32^ of TAS2R4, directly interacts with strychnine and is critical for strychnine activity on TAS2R46^8^.

The apo TAS2R4–miniG_gust_ structure lacks a preformed orthosteric vestibule, as many of the amino acids that interact with 3W in the 3W-bound TAS2R4 structure are not optimally positioned for 3W binding in the apo structure (Fig. 1g). Instead, the extracellular proximal regions of TM6 and TM7 shift toward TM3 and TM2, forming a stabilizing noncovalent network including hydrophobic contacts between T246^6.52^ and F88^3.32^, and Y250^6.56^ and V85^3.29^, as well as hydrogen bonds between Q249^6.55^ and N65^2.60^, and Y6^1.32^ and K262^7.35^, which must be broken for ligand binding (Fig. 1f–h). Upon activation, the receptor undergoes an extensive structural rearrangement (Fig. 1f). Viewing from the extracellular side, TM2 and TM3 of TAS2R4 superimpose relatively well between apo and 3W-bound states. The extracellular proximal regions of TM1, TM7, TM6, TM5 and TM4 “open up” in a way akin to petals opening on a flower, extending in a clockwise direction during the transition from the apo to the 3W-bound state (Fig. 1f). The largest difference between the apo and 3W-bound structures is a 10-Å outward movement of TM5 when measured at the Cα (α-carbon) of L175^5.37^. The extracellular regions of TM4 and TM5 become 6 Å closer when measured between the Cα of L150^4.63^ and L175^5.37^ (Fig. 1f). In this process, the agonist acts as a molecular bridge, restructuring TM orientations to stabilize the active state. This transition from a collapsed apo state to an expanded bound state demonstrates that the ligand does not simply occupy a pocket but actively drives the formation of the orthosteric site (Fig. 1f–h). This dramatic structural plasticity of the orthosteric vestibule observed in TAS2R4 indicates the existence of multiple ligand-specific conformational states, underlying the unique functional plasticity at least for a subgroup of TAS2Rs.

The comparison between apo and 3W-bound structures reveals significant structural propagation from the orthosteric site to the G protein. Upon 3W binding, the TAS2R4–miniG_gust_ interface has doubled, increasing from 1100 Å^2^ to 2441 Å^2^. While the intracellular regions of TM1–TM4 and TM7 remain stable, TM6 undergoes a unique 4-Å downward and 3-Å outward movement — distinct from the canonical class A GPCR bending — facilitated by lateral shifts (Fig. 1i)^11^.

These changes alter the cytoplasmic cavity and the engagement of the Gα_gust_ α5 helix. The α5 helix exhibits a 2.5 Å lateral shift toward TM6 and an 18° rotation toward TM5 (Fig. 1j). In the apo structure, S42^2.37^, S44^2.39^ and H284^8.47^ form hydrogen bonds with the carbonyls of D350, C351 and G352 of the α5 helix, K286^8.49^ forms a salt bridge with the C-terminal carboxylic acid of the α5 helix, and I110^3.54^ and A224^6.30^ form hydrophobic interactions with L348 and L353 of the α5 helix of Gα_gust_, together stabilizing the engaging position of α5 helix (Fig. 1k). In the 3W-bound structure, the downward movement of TM6 exposes A228^6.34^, which, along with I110^3.54^, forms hydrophobic interactions with L348 and L353 of the α5 helix, while A224^6.30^ forms new hydrophobic interactions with F354 of the Gα_gust_ α5 helix. Additionally, the outward movement of TM6 pulls the α5 helix, breaking the salt bridge with K286^8.49^ and resulting in the observed lateral and rotational movements of the α5 helix of Gα_gust_ (Fig. 1l).

Furthermore, all three intracellular loops (ICLs) undergo conformational changes to form direct contacts with miniG_gust_: ICL1 interacts with Gβ, while ICL2 and ICL3 contact Gα (Fig. 1m). Notably, the H39^1.65^–S309 bond (ICL1–Gβ) is essential for signaling (Fig. 1n; Supplementary Fig. S6c, d and Table S2). Among the three ICLs, ICL3 undergoes the most dramatic rearrangement, forming an extensive interface (Fig. 1n). Mutations within ICL3, such as W218^ICL3^A and R205^5.67^A, completely abolished signaling activity, confirming its role as a pivotal transduction element (Fig. 1n; Supplementary Fig. S6c, d and Table S2). Furthermore, the toggle switch Y242^6.48^ and the Y239^6.45^–D92^3.36^ hydrogen bond network facilitate a distinct activation path that bypasses several conserved motifs (like DRY and NPxxY) found in other GPCR classes (Fig. 1o; Supplementary Fig. S7 and Table S2)^8^.

By comparing our TAS2R4 structures with recently reported TAS2R46 and TAS2R14 structures, we uncovered a significant divergence in class T activation mechanisms. In the absence of exogenous ligand, TAS2R46 and TAS2R14 adopt a “pre-activated” state, whereas this behavior is not observed in TAS2R4. TAS2R46 is stabilized in an active-like conformation by its own ECL2, which occupies the orthosteric site (Supplementary Fig. S8a). Our mutation study on TAS2R46 (M172^ECL2^D) confirmed that disrupting this ECL2 interaction significantly reduces basal activity, supporting a “self-activation” hypothesis for TAS2R46 (Supplementary Fig. S8b and Table S3). For TAS2R14, an endogenous ligand cholesterol inserts deeply into the binding site and contributes to its basal activity (Supplementary Fig. S8c)^7^. Compared with TAS2R4, TAS2R46 and TAS2R14 undergo substantially fewer TM conformational rearrangements upon exogenous agonist binding (Supplementary Fig. S8a, d). Strychnine binding induces shifts in the ECLs of TAS2R46 (Supplementary Fig. S8a), whereas binding of compound 28.1 at the allosteric site of TAS2R14 elicits a conformational transition of TM6 from a curved to a linear conformation (Supplementary Fig. S8c, e). In contrast, TAS2R4 adopts a truly “collapsed” apo state that requires large-scale helical rearrangements for activation (Fig. 1f). Binding of 3W to TAS2R4 triggers 5.7–10-Å movements of TM5, TM6, and TM7, accompanied by a pronounced conformational rearrangement in ICL3 that mediates extensive interactions with Gα (Fig. 1f, i, j, n). This suggests that the hTAS2R family utilizes different activation strategies: some receptors (like TAS2R4) rely on extreme structural plasticity to detect ligands, some receptors (like TAS2R46) utilize structured loops to modulate basal sensitivity and ligand entry, while others (like TAS2R14) are co-activated by cholesterol acting as an orthosteric agonist and other bitter molecules serving as positive allosteric modulators.

Our dual-state cryo-EM analysis of TAS2R4 provides a comprehensive framework for understanding bitter taste perception. The hyper‑flexibility of the ECL2 region and TMs, as well as the specialized ICL3-mediated G protein coupling reveals a high degree of evolutionary specialization within class T GPCRs. These insights not only clarify the molecular principles of bitter signal transduction but also provide a structural basis for the development of taste modulators to enhance our understanding of taste transmission.

## Supplementary information

Supplementary Information

## Data Availability

Atomic coordinates and the cryo-EM density maps for structures of 3W–TAS2R4–miniG_gust_ and apo–TAS2R4–miniG_gust_ have been deposited in the RCSB Protein Data Bank (PDB) and the Electron Microscopy Data Bank (EMDB) with identification codes 25ST and 25SU, and EMD-63712 and EMD-63713, respectively. All other data are available in the manuscript or the supplementary materials. Reagents are available from the corresponding authors upon reasonable request.
