# Supplementary material for: Receptor hyper-flexibility of human bitter receptor TAS2R4 revealed by cryo-EM structures in apo and tripeptide-bound states
Source: Cell Discov. 2026 May 13;12:34. doi: 10.1038/s41421-026-00895-4 (PMC13172528; doi:10.1038/s41421-026-00895-4)
Supplement: Supplementary file 1 — Supplementary Information [file 41421_2026_895_MOESM1_ESM.pdf]

Supplementary Information for

Title: Receptor hyper-flexibility of human bitter receptor TAS2R4 revealed by cryo-EM structures in apo and tripeptide-bound states

Author: Yuxia Qian[1#], Ming Tan[2#], Zishao Ouyang[1#], Sheng Ye[1, 3, 4, 5*], Jian Wu[2*], Anna Qiao[1, 3, 4*]

Affiliations: [1] Tianjin Key Laboratory of Function and Application of Biological Macromolecular Structures, Faculty of Medicine, School of Life Sciences, Tianjin University, Tianjin, China. [2] Shanghai Institute of Precision Medicine, Ninth People's Hospital, Shanghai Jiao Tong University School of Medicine, Shanghai, China. [3] State Key Laboratory of Synthetic Biology, Tianjin University, Tianjin, China. [4] Frontiers Science Center for Synthetic Biology (Ministry of Education), Tianjin University, Tianjin, China. [5] Life Sciences Institute, Zhejiang University, Hangzhou, Zhejiang, China

[#] These authors contribute equally to this work

[*] Correspondence to:

Anna Qiao, School of Life Sciences, Tianjin University, 92 Weijin Road, Nankai District, Tianjin 300072, China; E-Mail: anna.qiao@tju.edu.cn

Jian Wu, Ninth People's Hospital, Shanghai Jiao Tong University School of Medicine, Shanghai 200125, China; E-Mail: wujian@shsmu.edu.cn

Sheng Ye, School of Life Sciences, Tianjin University, 92 Weijin Road, Nankai District, Tianjin 300072, China; E-Mail: sye@tju.edu.cn

This file includes:

Supplementary information, Figs. S1-S8

Supplementary information, Tables S1-S3

Materials and Methods

Supplementary information, Fig. S1

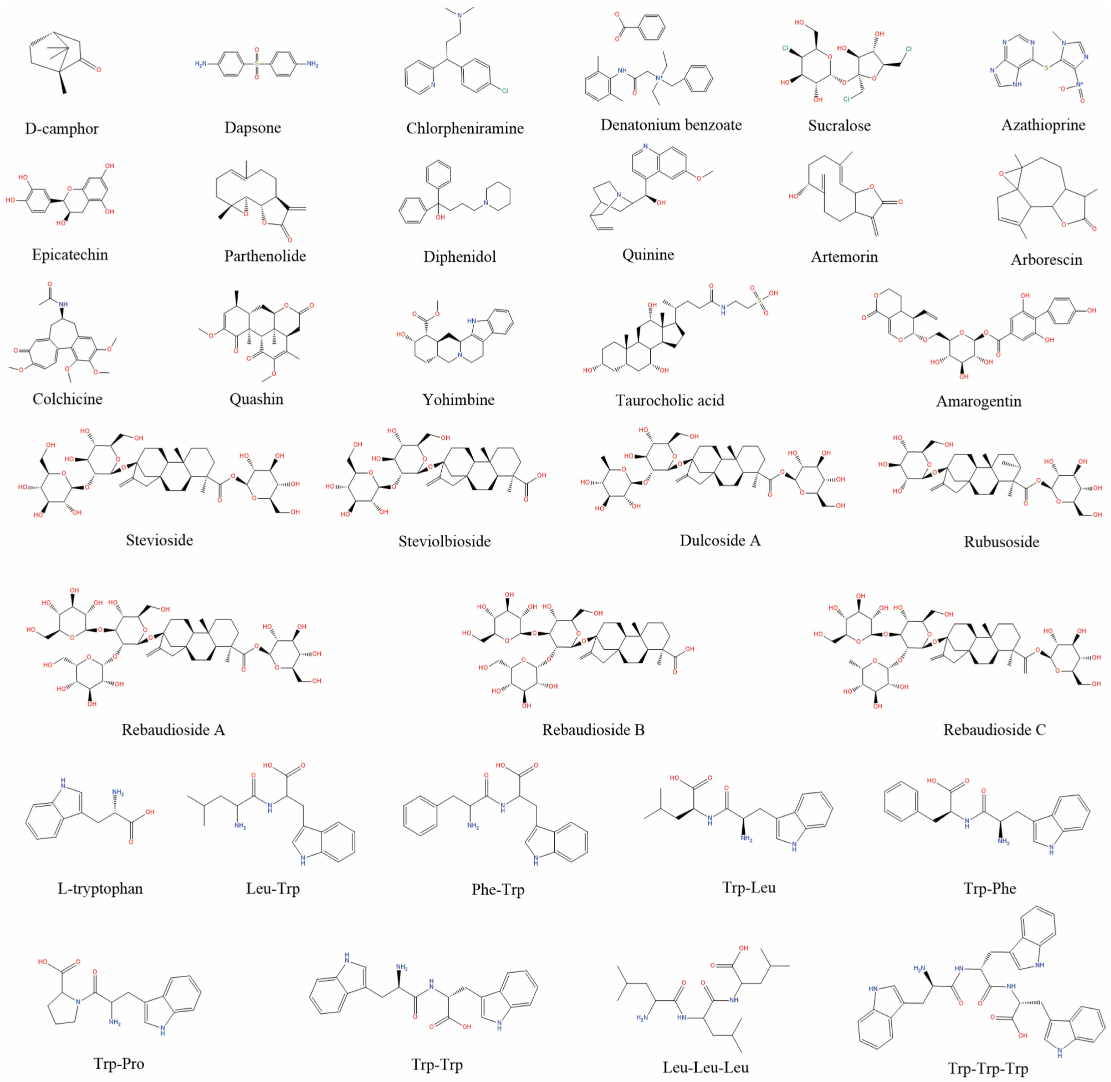

Supplementary information, Fig. S1. Bitter agonists of human TAS2R4. Chemical structures of 33 structurally diverse bitter agonists of human TAS2R4, including natural alkaloids, synthetic compounds, glycosides, and short peptides. Eight short-chain di- or tripeptides, the majority containing at least one tryptophan residue, are highlighted, with the tripeptide 3W identified as the most potent activator.

Supplementary information, Fig. S2

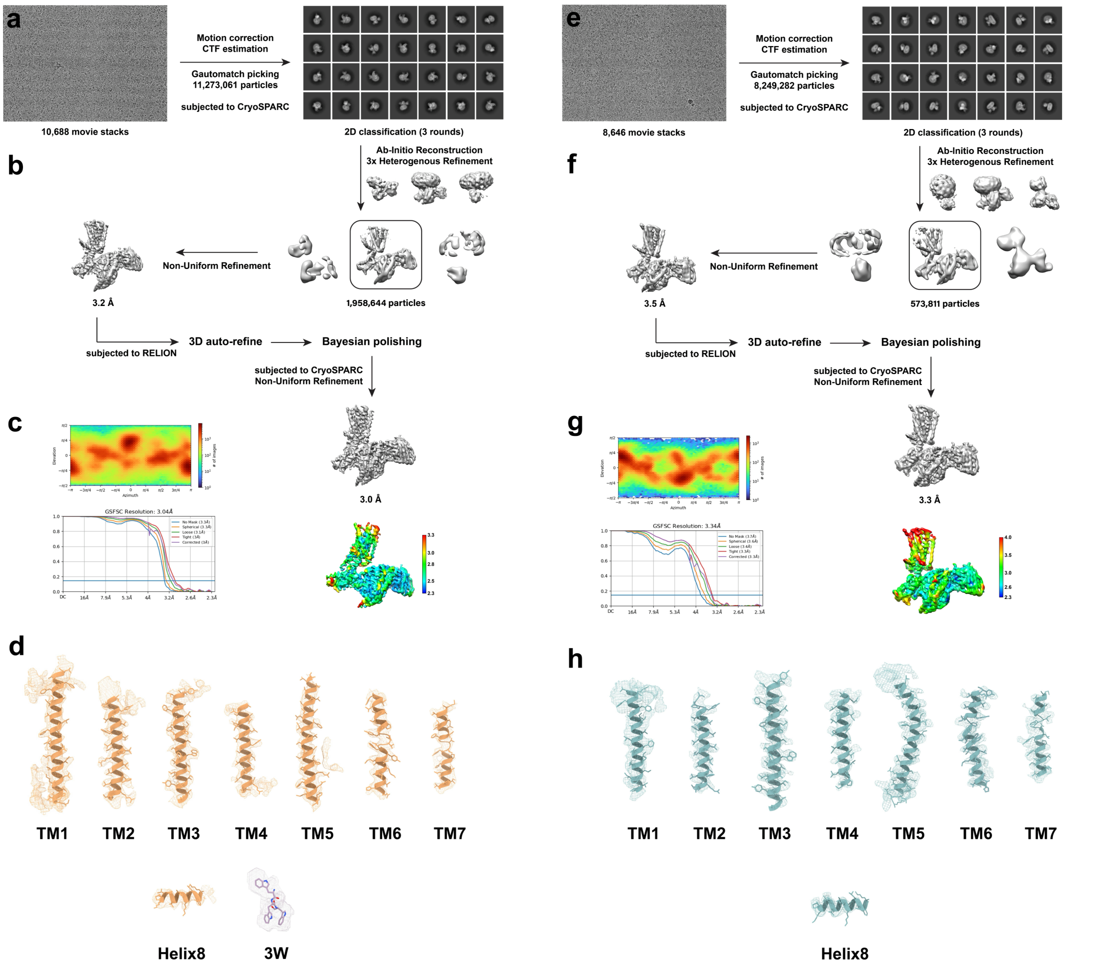

Supplementary information, Fig. S2. Cryo-EM data imaging and processing of the 3W-TAS2R4-miniG$_{gust}$ complex and apo-TAS2R4-miniG$_{gust}$ complex. (a–d) Results for the 3W-TAS2R4-miniG$_{gust}$ complex. **(e–h)** Results for the apo-TAS2R4-miniG$_{gust}$ complex. **(a, e)** Representative micrographs. **(b, f)** Cryo-EM data process workflow. **(c, g)** Fourier shell correlation (FSC) curves of the density maps. **(d, h)** Cryo-EM density of transmembrane helices of TAS2R4 and the cryo-EM density of ligand.

Supplementary information, Fig. S3

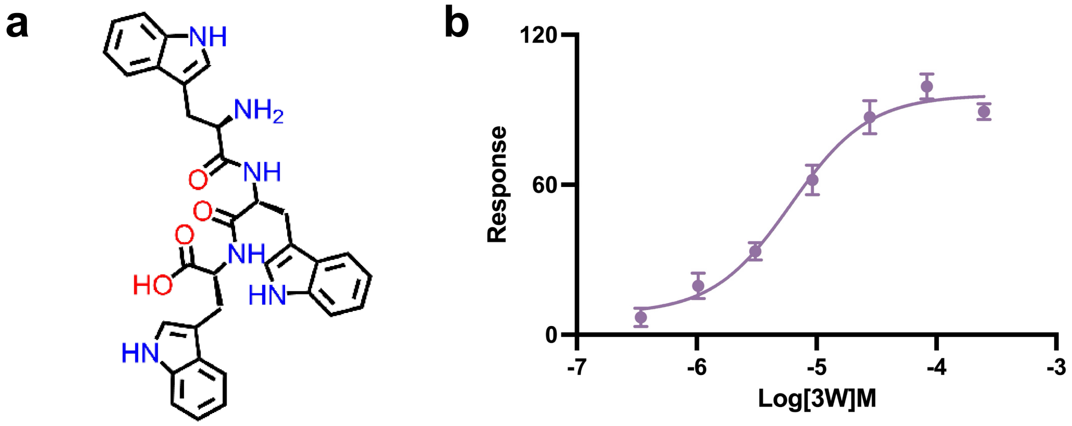

Supplementary information, Fig. S3. The chemical structure of 3W and its calcium flux curve. **(a)** Chemical structure of the tripeptide agonist 3W. **(b)** Concentration-dependent intracellular Ca^{2+} flux signal induced by 3W activation of TAS2R4, with EC50 = 7.5 ± 0.1 μM. Data are represented as mean ± S.E. (n = 3).

Supplementary information, Fig. S4

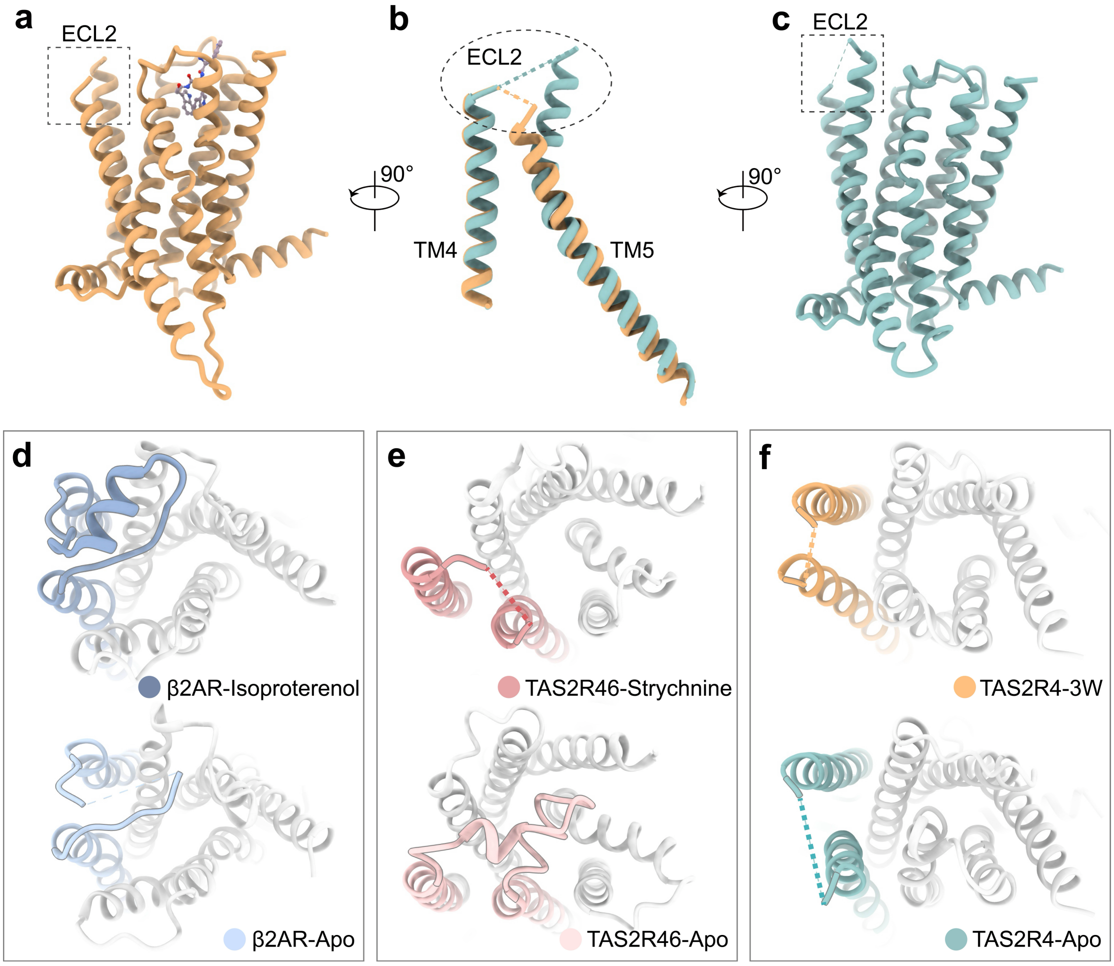

Supplementary information, Fig. S4. Flexibility of extracellular loop 2 (ECL2) in TAS2R4. **(a)** Cartoon representation of TAS2R4 in the 3W-bound state. **(b)** Overlay of ECL2 structures in TAS2R4, highlighting its flexibility in both apo and 3W-bound states. **(c)** Cartoon representation of TAS2R4 in the apo state. **(d–f)** Structural representations of ECL2 in β2AR **(d)**, TAS2R46 **(e)**, and TAS2R4 **(f)**.

Supplementary information, Fig. S5

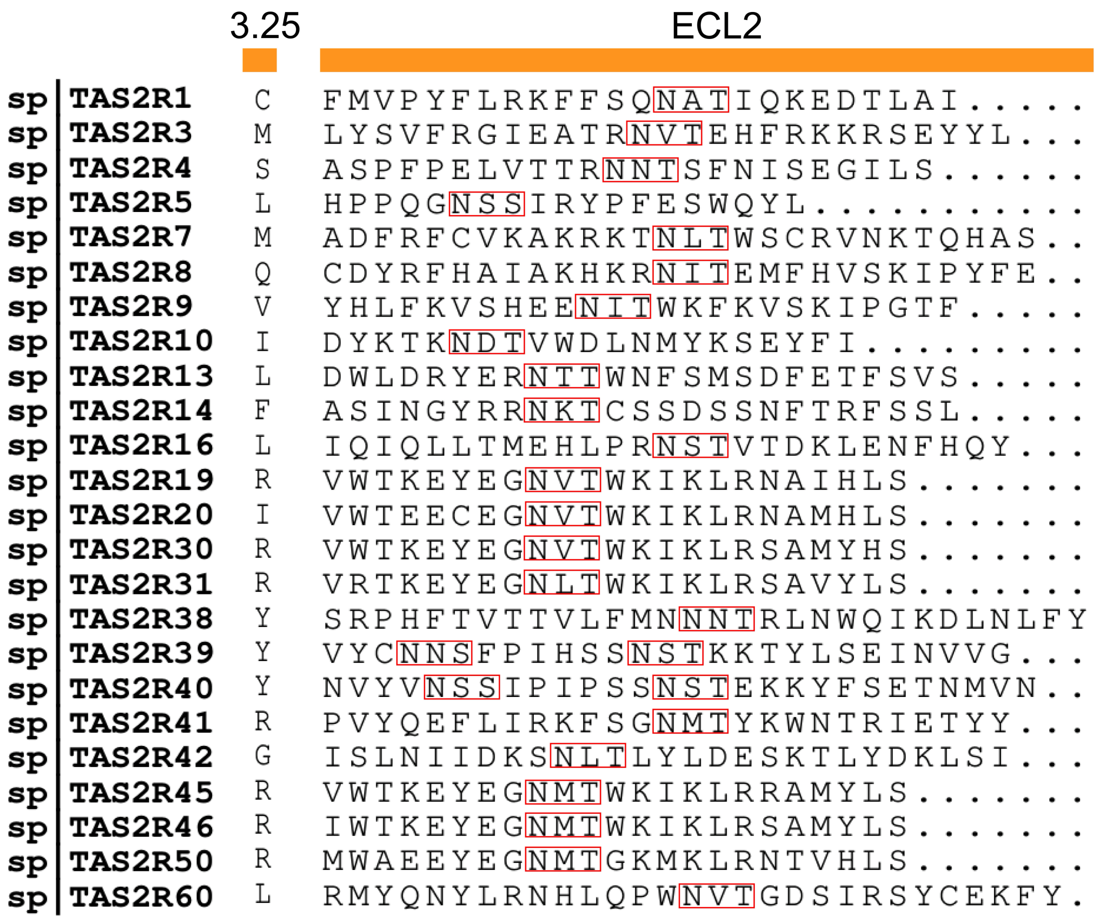

Supplementary information, Fig. S5. Sequence alignment of TAS2R receptors highlighting position 3.25 and ECL2. TAS2Rs lack the conserved $C^{3.25}$–ECL2 disulfide bond found in class A GPCRs but contain a conserved Asn-Xaa-Ser/Thr glycosylation motif in ECL2, critical for receptor function.

Supplementary information, Fig. S6

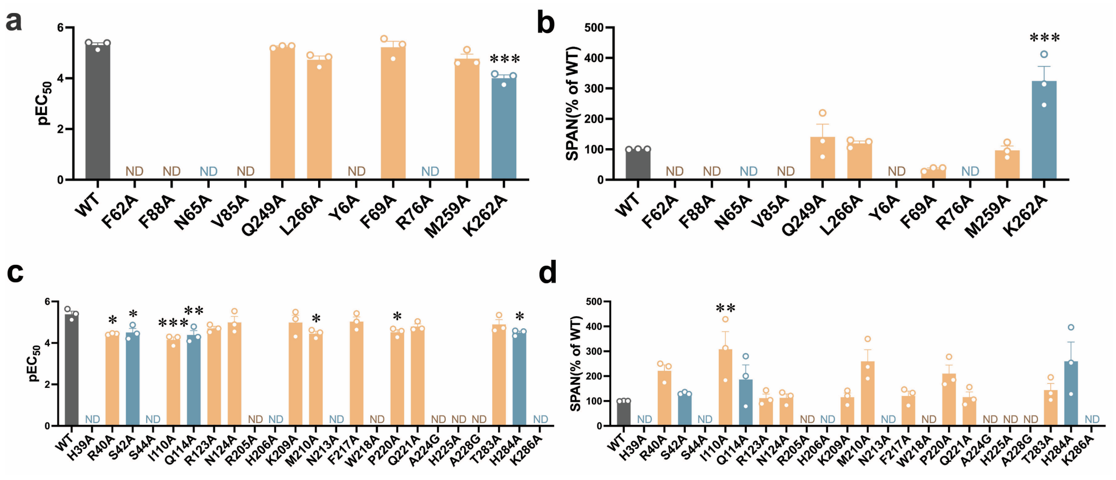

Supplementary information, Fig. S6. Ca^{2+} flux signals induced in wild-type TAS2R4 and mutants by 3W. **(a, b)** Ca^{2+} flux signals induced in wild-type TAS2R4 and mutants in binding pocket by 3W. **(c, d)** Ca^{2+} flux signals induced in wild-type TAS2R4 and mutants of key residues involved in G protein interaction. Data are mean ± s.e.m. from at least three independent experiments (*n* = 3). *P<0.1; **P<0.01; ***P<0.001 by one-way ANOVA followed by Dunnett's post-test, compared with the response of the WT. WT, hydrophobic interaction residues, and hydrogen bonding residues are shown in grey, orange and blue, respectively.

Supplementary information, Fig. S7

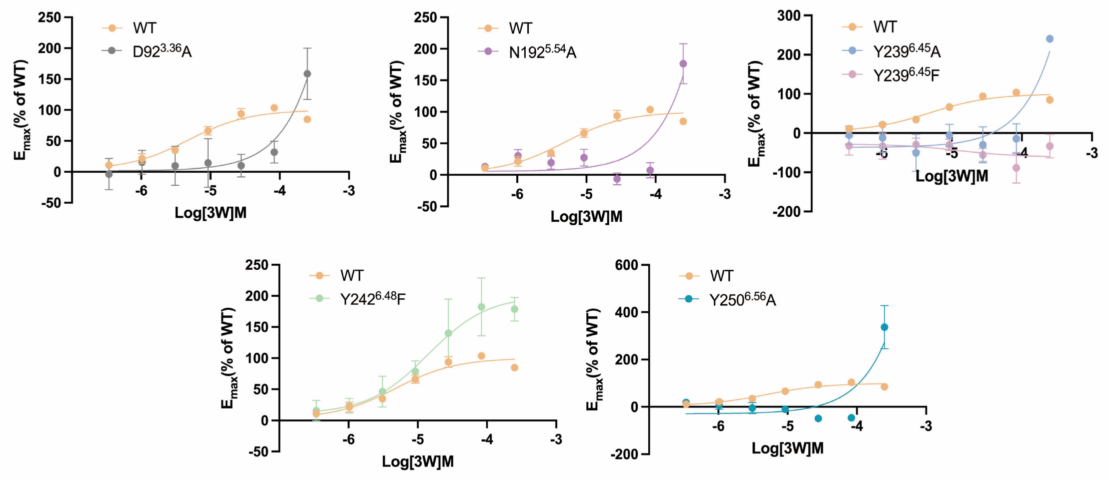

Supplementary information, Fig. S7. Effects of key mutations on TAS2R4-miniGα_{gust} activation by 3W. Data are mean ± s.e.m. of three independent experiments performed in triplicate.

Supplementary information, Fig. S8

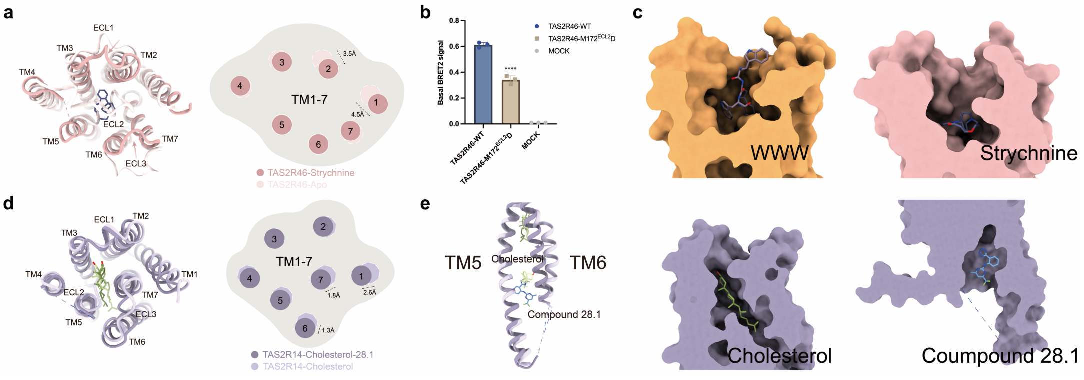

Supplementary information, Fig. S8. Structural comparison of TAS2R4-WWW with TAS2R14 and TAS2R46. **(a)** Views from the extracellular side: structural rearrangements of transmembrane helices and ECLs following activation of TAS2R46 (PDB: 7XP4, 7XP6). **(b)** Basal BRET2 signals were calculated by subtracting the BRET2 basal ratio of the MOCK group from the BRET2 basal ratio of TAS2R46-WT and TAS2R46-M172^{ECL2}D groups, respectively. Data are mean ± s.e.m. from at least three independent experiments (n = 3). **(c)** Sectional views of ligand-binding sites in receptors: WWW in active-state TAS2R4, strychnine in active-state TAS2R46 (PDB: 7XP6), cholesterol and compound 28.1 in active-state TAS2R14 (PDB: 8YKY). **(d)** Views from the extracellular side: structural rearrangements of transmembrane helices following activation of TAS2R14 (PDB: 8XQT, 8YKY). **(e)** Conformational transition of TM6 change from arc to linear after binding of compound 28.1 in TAS2R14 (PDB: 8XQT, 8YKY).

Supplementary information, Table S1. Cryo-EM data collection, refinement and validation statistics

	**3W-TAS2R4-miniG$_{gust}$ complex** (EMD-63712) (PDB 25ST)	**apo-TAS2R4-miniG$_{gust}$ complex** (EMD-63713) (PDB 25SU)
**Data collection and processing**		
Magnification	81,000	81,000
Voltage (kV)	300	300
Electron exposure (e^-/Å^2)	50	50
Defocus range (μm)	-1.5 ~ -2.0	-1.5 ~ -2.0
Pixel size (Å)	1.1	1.1
Symmetry imposed	C1	C1
Initial particle projections (no.)	11,273,061	8,249,282
Final particle projections (no.)	1,958,644	573,811
Map resolution (Å)	3.0	3.3
FSC threshold	0.143	0.143
Map resolution range (Å)	2.43 ~ 5.72	2.4 ~ 7.29
**Refinement**		
Initial model used	AlphaFold2	AlphaFold2
Model resolution (Å)	3.3	3.5
FSC threshold	0.5	0.5
Map sharpening B factor (Å^2)	-137.1	-160.5
Model composition		
Non-hydrogen atoms	8,992	8989
Protein residues	1,147	1,150
Ligand	0	0
B-factors (Å^2)		
Protein	54.54	51.84
Ligand	-/-	-/-
R.m.s. deviations		
Bond lengths (Å)	0.002	0.002
Bond angles (°)	0.583	0.557
Validation		
MolProbity score	1.85	1.71
Clashscore	6.69	7.74
Rotamer outliers (%)	2.40	1.40
Ramachandran plot		
Favored (%)	96.82	97.00
Allowed (%)	3.09	3.00
Disallowed (%)	0.09	0.00

Supplementary information, Table S2. Tripeptide 3W-induced intracellular calcium mobilization of wild-type (WT) and mutant TAS2R4.

Mutants	EC_{50} (μM)	pEC50±SEM[a]	SPAN (% of WT) [a, b]	n[c]	Expression (% WT) [a]
Wild type	6.11	5.21±0.09	100±8	9	100
$Y6^{1.32}A$	nd	nd	nd	3	52±13
$H39^{1.65}A$	nd	nd	nd	3	99±19
$R40^{ICL1}A$	36.6	$4.44\pm0.19^{*}$	221±25	3	138±27
$S42^{2.37}A$	26.2	$4.58\pm0.23^{*}$	124±16	3	117±23
$S44^{2.39}A$	nd	nd	nd	3	84±11
$F62^{2.57}A$	nd	nd	nd	3	144±16
$N65^{2.60}A$	nd	nd	nd	3	96±17
$F69^{2.64}A$	6.79	5.17±0.35	33±6	3	133±12
$R76^{ECL1}A$	nd	nd	nd	3	90±22
$V85^{3.29}A$	nd	nd	nd	3	88±13
$F88^{3.32}A$	nd	nd	nd	3	105±22
$D92^{3.36}A$	nd	nd	nd	3	78±15
$I110^{3.54}A$	66.3	$4.18\pm0.28^{***}$	$288\pm58^{**}$	3	96±11
$Q114^{ICL2}A$	54.6	$4.26\pm0.38^{**}$	185±48	3	47±8
$R123^{ICL2}A$	20.6	4.69±0.38	112±23	3	86±17
$N124^{4.37}A$	9.85	5.00±0.36	105±19	3	124±10
$N192^{5.54}A$	nd	nd	nd	3	112±9
$R205^{5.67}A$	nd	nd	nd	3	104±12
$H206^{5.68}A$	nd	nd	nd	3	79±7
$K209^{5.71}A$	10.7	4.97±0.34	106±18	3	131±17
$M210^{5.72}A$	37.6	$4.42\pm0.18^{*}$	255±27	3	116±23
$N213^{5.75}A$	nd	nd	nd	3	141±11
$F217^{ICL3}A$	13.0	4.89±0.21	117±13	3	130±15
$W218^{ICL3}A$	nd	nd	nd	3	61±5
$P220^{6.26}A$	26.4	$4.58\pm0.16^{*}$	206±19	3	$220\pm23^{***}$
$Q221^{6.27}A$	17.1	4.77±0.21	114±13	3	147±18
$A224^{6.30}G$	nd	nd	nd	3	168±22
$H225^{6.31}A$	nd	nd	nd	3	152±23
$A228^{6.34}G$	nd	nd	nd	3	169±28
$Y239^{6.45}A$	nd	nd	nd	3	69±13
$Y239^{6.45}F$	nd	nd	nd	3	65±7
$Y242^{6.48}F$	14.0	4.85±0.33	190±33	3	106±19
$Q249^{6.58}A$	5.86	5.23±0.32	141±23	3	63±15
$Y250^{6.56}A$	nd	nd	nd	3	150±7
$M259^{7.32}A$	17.6	4.76±0.30	94±15	3	94±9
$K262^{7.35}A$	70.5	$4.15\pm0.24^{***}$	$323\pm58^{***}$	3	99±23
$L266^{7.39}A$	20.2	4.70±0.35	118±22	3	97±23
$H284^{8.47}A$	27.8	$4.56\pm0.31^{*}$	257±45	3	95±15
$K286^{8.49}A$	nd	nd	nd	3	81±12

[a]Data are shown as mean±SEM from at least three independent experiments performed in technical triplicate. $^{*}P<0.1$; $^{**}P<0.01$; $^{***}P<0.001$ by one-way ANOVA followed by Dunnett's post-test, compared with the response of the WT.

[b]The span is defined as the window between the maximal 3W response (E_{max}).

[c]Sample size; the number of independent experiments performed in technical triplicate.

Supplementary information, Table S3. Basal BRET2 signal of wild-type (WT) and mutant TAS2R46.

Mutants	Basal signal[a]	n[b]	Expression (% WT)[a]
Wild type	0.61±0.01	3	100
M172^{ECL2}D	0.34±0.02****	3	91±20

[a]Data are shown as mean±SEM from at least three independent experiments performed in technical triplicate. ****P<0.0001 by one-way ANOVA followed by Dunnett's post-test, compared with the response of the WT.

[b]Sample size; the number of independent experiments performed in technical triplicate.

Materials and Methods

Construct cloning

To obtain TAS2R4-miniGα_{gust} complex, the human wild-type TAS2R4 gene was cloned into a modified pFastBac1 vector with the haemagglutinin signal peptide (HA) and a Flag tag (DYKDDDD) at N terminus, a PreScission protease site and a twin-Strep-tag (WSHPQFEK-GGGSGGGSGGSA-WSHPQFEK) at C terminus.

To modify miniGα_{gust}, GαAH domain was deleted and replaced by a linker (GGGGGGGG), and 57 amino acids at N terminus was replaced with G_{i1}, and 6 mutants G42D, E43N, G217D, A226D, V332A, V335I were added.

Human RIC8A, G$\beta_3\gamma_{13}$, and a single chain antibody scFv16[1] were cloned into the pFastBac vector.

Insect cell expression

Coexpression of receptor TAS2R4, miniGα_{gust}, RIC8A and G$\beta_3\gamma_{13}$ were conducted in HighFive insect cells (Invitrogen) using the Bac-to-Bac Baculovirus Expression System (Invitrogen). Cells were grown to a density of 2.5×10^6 cells per milliliter and then coinfected with the high-titer recombinant baculovirus at a multiplicity of infection (MOI) ratio of 1:1:1:1. The cells were collected by centrifugation after transfection for 48 hours at 27 °C, and stored at -80 °C until use.

Purification of apo/3W-TAS2R4-miniGα_{gust} complexes

Cells were thawed and suspended in a buffer containing 20 mM HEPES (pH 7.5), 50 mM NaCl, 2 mM $MgCl_2$ and EDTA-free protease inhibitor cocktail tablets (Roche). To facilitate the formation of the TAS2R4-miniGα_{gust} complex, 10 μg/ml ScFv16 prepared as previously reported[2], 25 mU/mL Apyrase (New England BioLabs) and 300 μM 3W were added to the cell suspension. The mixture was incubated at 16 °C for 1 hour. Following incubation, membrane pellets were collected by ultracentrifugation at 38,000 rpm for 30 minutes. The complexes were then extracted from the membrane with an extraction buffer containing 20 mM HEPES (pH 7.5), 150 mM NaCl, 2 mM $MgCl_2$, 0.5% (w/v) lauryl maltose neopentyl glycol (L-MNG, Anatrace), 0.025% (w/v) cholesteryl hemisuccinate (CHS, Anatrace), 25 mU/ml Apyrase, and 1 mM 3W. After a 2-hour incubation period at 4 °C, the supernatant was isolated by ultracentrifugation at 38,000 rpm for 30 minutes, and subsequently incubated overnight with Strep-Tactin®XT 4Flow® resin (IBA) at 4 °C. The

resin was washed with 20 column volumes of wash buffer (25 mM HEPES, pH 7.5, 150 mM NaCl, 2 mM $MgCl_2$, 0.01% (w/v) L-MNG, 0.0005% (w/v) CHS, and 300 μM 3W), followed by elution with 10 column volumes of elution buffer (25 mM Tris, pH 7.5, 150 mM NaCl, 2 mM $MgCl_2$, 50 mM Biotin, 0.01% (w/v) L-MNG, 0.0005% (w/v) CHS, and 1 mM 3W). The eluted complexes were further purified by size-exclusion chromatography using a Superdex 200 Increase 10/300 column (GE Healthcare) pre-equilibrated with 20 mM HEPES (pH 7.5), 150 mM NaCl, 2 mM $MgCl_2$, 0.01% (w/v) L-MNG, 0.0005% (w/v) CHS, and 300 μM 3W. The same protocol was used to purify the apo form, omitting the addition of an agonist. Finally, the purified complexes were concentrated to 3-4 mg/ml using a 100-kDa molecular weight cut-off concentrator (Millipore) and analyzed by SDS-PAGE.

Cryo-EM grid preparation and data collection

For cryo-EM grid preparation, 3.5 μL of the purified complex sample was applied onto freshly glow-discharged holey carbon grids (Quantifoil R1.2/1.3, Au 300 mesh). The grids were blotted for 2.5 seconds under 100% humidity at 4 °C and then rapidly plunge-frozen in liquid ethane using a Thermo Fisher Vitrobot Mark IV. Cryo-EM data were collected using EPU software on a 300 kV Titan Krios G3i electron microscope (FEI) equipped with a K3 Summit direct electron detector and a Gatan Quantum energy filter. Movie stacks were acquired at a magnification of 81,000×, corresponding to a pixel size of 1.1 Å. Each micrograph was dose-fractionated into 32 frames over 3.72 seconds, resulting in a total accumulated dose of 50 $e^{-}Å^{-2}$. The defocus range was set between -1.5 and -2.0 μm.

Cryo-EM data processing

For the 3W- and apo-TAS2R4-miniGα_{gust} complex, a total of 10,688 and 8,646 movies respectively underwent beam-induced motion correction and dose-weighting using RELION MotionCor2[3]. Contrast transfer function estimation was performed with Gctf[4]. Images with poor statistics were discarded. Particle picking was carried out using Gautomatch-v0.56 (https://github.com/JackZhang-Lab/Gautmatch) and a subset of particles after 2D classification was utilized to generate initial references using the Ab-Initio reconstruction module in CryoSPARC[5]. For each full dataset, a total of 11,273,061 and 8,249,282 particles were extracted

respectively and subjected to several rounds of 2D classification and heterogeneous refinement to exclude poor particles. Subsequently, Bayesian polishing was performed in RELION on the selected particles in the best classes. Finally, a subset of 1,958,644 and 573,811 polished particles then subjected to CryoSPARC for further non-uniform refinement followed by local resolution estimation with the final resolution of 3W- and apo-TAS2R4-miniGα_{gust} complex at 3.0 Å and 3.3 Å, respectively.

Model building and refinement

For the structure of the 3W- and apo-TAS2R4-miniGα_{gust} complex, the initial template of TAS2R4 was generated using AlphaFold2[6]. 3W coordinates and geometry restrain were generated using Phenix[7], elbow. Models was docked into the density map using UCSF Chimera[8], followed by iterative manual adjustments in COOT[9]. The model was further refined by real-space refinement using Phenix.

Intracellular calcium mobilization assay

We constructed stable transfected cell lines expressing G$\alpha_{16/gust44}$[10] using the Flp-In™ T-REx™ 293 cell line (Thermo Fisher, R78007). The cells were co-transfected with the corresponding plasmids, pcDNA™5/FRT/TO (Thermo Fisher, V652020) and pOG44 (Thermo Fisher, V600520), following the manufacturer's instructions. The resulting $G_{\alpha16/gust44}$ cell lines were maintained in complete DMEM supplemented with 10% (v/v) fetal bovine serum ((FBS Gibco, 10270106)) and 1% penicillin-streptomycin (Gibco, 15140122). For experiments, cells were seeded at a density of 2×10^6 cells per well in 6 cm culture dishes and incubated overnight at 37 °C.

Transient transfection was performed using 4 μg of either wild-type TAS2R4 or mutant TAS2R4 plasmid DNA with Polyethylenimine Linear (PEI, MW 40000, YEASEN, 40816ES03). After 6–8 hours of transfection, the medium was replaced with complete DMEM containing 2 μg/ml puromycin. At 24 hours post-transfection, the cells were diluted in complete DMEM supplemented with 2 μg/ml doxycycline (Solarbio, D8960) and seeded at a density of 80,000 cells per well in a black clear-bottom 96-well plate (Corning®, 3340) pre-coated with D-Lysine homopolymer hydrobromide (Sigma, P7886). After an additional 24 hours, the medium was replaced with 40 μL of HBSS buffer containing 20 mM HEPES (pH 7.4), 4 mM Fluo-8H™, and

2.5 mM probenecid. The plate was incubated at 37 °C for 40 minutes to allow dye loading. Fluorescence measurements were performed using a FlexStation 3 Multi-Mode Microplate Reader at 37 °C. Baseline readings were recorded in 100 µL of HBSS buffer containing 20 mM HEPES (pH 7.4). At 20 seconds, the instrument automatically added 50 µL of a triple concentration of 3W, and readings were continued for 120 seconds, with data collected every two seconds. The resulting data were analyzed using nonlinear regression analysis in GraphPad Prism 9.0.

Wild-type TAS2R4 and mutant TAS2R4 were cloned into the pcDNA3.1 vector, with HA and Flag tags incorporated at the N-terminus of the receptors (as described above). An empty pcDNA3.1 vector was used as a negative control. The cell surface expression levels of the receptors were detected using a monoclonal anti-FLAG M2-fluorescein isothiocyanate antibody (Sigma-Aldrich).

BRET2 assays

BRET2 probes were prepared by cloning TAS2R46, $G\alpha_{gust}$-RLuc8, $G\beta_3$, and GFP2-$G\gamma_1$ into the pcDNA3.1 vector based on the TRUPATH assay resources and protocol[11]. For the BRET2 assays, 3 µg of plasmid DNA, in a ratio of TAS2R46: $G\alpha_{gust}$-RLuc8: $G\beta_3$: GFP2-$G\gamma_1$ = 1:1:1:1, were transiently co-transfected into HEK293 cells using PEI. After 24 hours, cells were seeded into 96-well plates at a density of 8×10^4 cells per well in 100 µL of culture medium. After another 24 hours, the medium was removed and replaced with 90 µL of HBSS buffer containing 20 mM HEPES (pH 7.4) for a 5-minute incubation. Subsequently, 10 µL of the luciferase substrate coelenterazine 400h (5 µM) was added, and BRET signals were immediately measured using a PerkinElmer microplate reader. BRET signals were calculated as the emission ratio of light at 525 nm to 400 nm.

References

1 Krishna Kumar, K. *et al.* Structure of a Signaling Cannabinoid Receptor 1-G Protein Complex. *Cell* **176**, 448-458 e412 (2019). https://doi.org:10.1016/j.cell.2018.11.040

2 Koehl, A. *et al.* Structure of the micro-opioid receptor-G(i) protein complex. *Nature* **558**, 547-552 (2018). https://doi.org:10.1038/s41586-018-0219-7

3 Zheng, S. Q. *et al.* MotionCor2: anisotropic correction of beam-induced motion for improved cryo-electron microscopy. *Nat Methods* **14**, 331-332 (2017). https://doi.org:10.1038/nmeth.4193

4 Zhang, K. Gctf: Real-time CTF determination and correction. *J Struct Biol* **193**, 1-12 (2016). https://doi.org:10.1016/j.jsb.2015.11.003

5 Punjani, A., Rubinstein, J. L., Fleet, D. J. & Brubaker, M. A. cryoSPARC: algorithms for rapid unsupervised cryo-EM structure determination. *Nat Methods* **14**, 290-296 (2017). https://doi.org:10.1038/nmeth.4169

6 Jumper, J. *et al.* Highly accurate protein structure prediction with AlphaFold. *Nature* **596**, 583-589 (2021). https://doi.org:10.1038/s41586-021-03819-2

7 Adams, P. D. *et al.* PHENIX: a comprehensive Python-based system for macromolecular structure solution. *Acta Crystallogr D Biol Crystallogr* **66**, 213-221 (2010). https://doi.org:10.1107/S0907444909052925

8 Pettersen, E. F. *et al.* UCSF Chimera--a visualization system for exploratory research and analysis. *J Comput Chem* **25**, 1605-1612 (2004). https://doi.org:10.1002/jcc.20084

9 Emsley, P. & Cowtan, K. Coot: model-building tools for molecular graphics. *Acta Crystallogr D Biol Crystallogr* **60**, 2126-2132 (2004). https://doi.org:10.1107/S0907444904019158

10 Ueda, T., Ugawa, S. & Shimada, S. Functional interaction between TAS2R receptors and G-protein alpha subunits expressed in taste receptor cells. *Chem Senses* **30** **Suppl** **1**, i16 (2005). https://doi.org:10.1093/chemse/bjh090

11 Olsen, R. H. J. *et al.* TRUPATH, an open-source biosensor platform for interrogating the GPCR transducerome. *Nat Chem Biol* **16**, 841-849 (2020). https://doi.org:10.1038/s41589-020-0535-8
